# Machine-Learning-Based Depression Detection Model from Electroencephalograph (EEG) Data Obtained by Consumer-Grade EEG Device

**DOI:** 10.3390/brainsci14111107

**Published:** 2024-10-30

**Authors:** Kei Suzuki, Tipporn Laohakangvalvit, Midori Sugaya

**Affiliations:** College of Engineering, Shibaura Institute of Technology, Research Building #14A32, 3-7-5 Toyosu, Koto-ku, Tokyo 135-8548, Japan; nb23108@shibaura-it.ac.jp (K.S.); doly@shibaura-it.ac.jp (M.S.)

**Keywords:** electroencephalography, depression, machine learning

## Abstract

**Background/Objectives:** There have been attempts to detect depression using medical-grade electroencephalograph (EEG) data based on a machine learning approach. EEG has garnered interest as a method for assessing brainwaves by attaching electrodes to the scalp to obtain electrical activity in the brain. Recently, machine learning has been applied to the EEG data to detect depression, with encouraging results. Specifically, studies using medical-grade EEG data have shown that depression can be accurately detected. However, there is a need to expand the range of applications by achieving a score with machine learning using simpler consumer-grade brain wave sensors. At present, a sufficient score has not been achieved.; **Methods:** To improve the score of depression detection, we quantified various EEG indices to train models such as power spectrum, asymmetry, complexity, and functional connectivity. In addition, feature selection was performed to ensure that the model learns only promising EEG indices for depression detection. The feature selection methods were Light Gradient Boosting Machine (LightGBM) feature importance, mutual information, ReliefF and ElasticNet coefficients. The selected EEG indices were learned by the LightGBM model, which is reported to be as accurate as the latest deep learning models. In cross-validation, the independence of test and training data was ensured to avoid excessively calculated score; **Results**: The results showed that the Macro F1 score was 91.59%, suggesting that a consumer-grade EEG can detect depression. In addition, analysis of the EEG indices selected by feature selection indicated that the Macro F1 score was about 80% for single EEG indices such as differential entropy in the frequency band β and functional connectivity in the left frontal region in the frequency band 1–128 Hz; **Conclusions**: Although the data were obtained from a consumer-grade EEG, the results suggest that these EEG indices are promising for detection depression.

## 1. Introduction

In recent years, the number of patients with mental disorders has increased. According to a report by the World Health Organization (WHO), as many as 264 million people bears some form of mental disorder. In addition, approximately 30% of the world’s population are expected to suffer from some mental disorders at some point during their lives [1]. In particular, the number of depressed patients has been reported to be increasing since the pandemic of the new coronavirus [2]. Hence, more appropriate diagnoses are needed to provide health care and treatment for this growing number of depressed patients.

However, in current diagnostic methods, there are challenges of inconsistency and instability in diagnostic concordance and reliability [3]. Current diagnostic methods depend on interviews conducted by clinicians and questionnaires answered by patients. These follow the *Diagnostic and Statistical Manual of Mental Disorders, Fifth Edition* (DSM-5) [4], which is the international standard for the diagnosis of mental disorders. However, the definitions of symptoms and other criteria presented in the diagnostic criteria are not always clear [3]. Therefore, it has been pointed out that the diagnosis can be influenced by the doctor’s subjectivity and skill, as well as the patient’s subjectivity [5]. The international standard, the DSM-5, was also designed to improve the reliability of diagnoses of mental disorders. However, the challenge remains that diagnostic criteria do not include objective biological assessment [3]. Therefore, the clinician’s diagnosis needs to be supported by objective assessments rather than interviews, as well as by the subjectivity of the clinician and patient.

One of the objective biological assessments is the analysis of data obtaining brain activity using tools such as Positron Emission Tomography (PET), functional Magnetic Resonance Imaging (fMRI), electroencephalograph (EEG), and other methods [6]. Among those methods, EEG is non-invasive, easy to record, and relatively inexpensive. Therefore, the use of EEG is preferred as an objective assessment, and its use in the assessment of depression is being attempted as research [7]. In addition, there are several studies that apply machine learning and deep learning to detect brain wave patterns as the signs of depression and compare between depressed patients and healthy controls [8,9].

There are several related studies that have applied machine learning and proposed EEG-based depression detection models with high score. Movahed et al. [10] quantified the features of EEG data, for example, statistical values including minimum and maximum values, power in several frequency bands from frequency analysis, and nonlinear indices including self-similarity and irregularities in EEG signals. Then, they used those EEG features to train a machine learning model using a Support Vector Machine (SVM). As a result, they achieved an accuracy of 99% in classifying depressed patients from healthy controls.

Khan et al. [11] created an image of wavelet coherence quantifying the statistical dependence of EEG data that can be obtained from multiple locations of electrodes. Then, they constructed a model by learning the images to a Convolutional Neural Network (CNN) algorithm. In evaluating the model, they devised the score evaluation method as follows: If the model correctly predicted more than half of the images extracted from the EEG data of the same participant, it was considered a correct detection of whether the participant was a depressed patient or a healthy control. As a result, they achieved 100% accuracy.

Avots et al. [12] quantified EEG features including relative power of frequency bands, power variation values, and spectral asymmetry by a frequency analysis of EEG data. In addition, they calculated the nonlinear indices of entropy and the self-similarity of EEG data. Then, they selected EEG features to train the models using several algorithms such as SVM, Linear Discriminant Analysis (LDA), Naive Bayes (NB), and k-Nearest Neighbor (kNN). As a result, they achieved an accuracy of 80–95% in classifying depressed patients and healthy controls.

These previous studies suggest that it is possible to distinguish depressed patients from healthy controls with a high score. However, these studies achieve the high score results by using medical-grade EEG devices rather than consumer-grade EEG devices. Clinicians working in large hospitals may be able to use expensive EEG devices. However, it may not be suitable for clinicians working in small clinics or for the individuals to monitor their own health at home. Therefore, in this study, to facilitate the use of EEG-based depression detection techniques, we will obtain EEG data using a consumer-grade EEG device, not an expensive medical-grade EEG device. We obtained EEG data in a resting state with eyes closed. This is because, in recent years, it has been indicated that EEG in the resting state with eyes closed is useful for estimating mental disorders [13]. Then, by selecting EEG features promising for detection depression and the EEG features learned by model, we aimed to construct a model to detect depressed patients from healthy controls. In this model construction, the objective variable was binary, indicating whether the patient was depressed or healthy, and the explanatory variables were EEG features.

The structure of this paper is as follows: Section 2 describes the data collection method; Section 3 describes the preprocessing method of the collected EEG data and the construction of the dataset; Section 4 describes the methods for machine learning model, cross-validation, evaluation scores, and feature selection; Section 5 describes the results and the evaluation of the selected features and the constructed models; Section 6 discusses our results and future works, and Section 7 concludes the paper.

## 2. Data Collection

### 2.1. Participants

We recruited healthy controls and participants with mental and developmental disorders to obtain their EEG data. The participants were those who had individually gone to the hospital and been diagnosed with these disorders before being recruited for the experiment. From these participants, only depressed patients’ and healthy controls’ data were selected for further analysis. There were 8 patients diagnosed with depression, including 6 males and 2 females. The average and standard deviation of the age of the depressed patients were 35.9 and 7.93. There were nine males as healthy controls. The average and standard deviation of the ages of the healthy controls were 21.3 and 0.48. The experiment was approved by the Ethics Review Committee of Shibaura Institute of Technology. All participants signed an informed consent form before the experiment.

### 2.2. Devices for Data Collection

**Figure 1:**
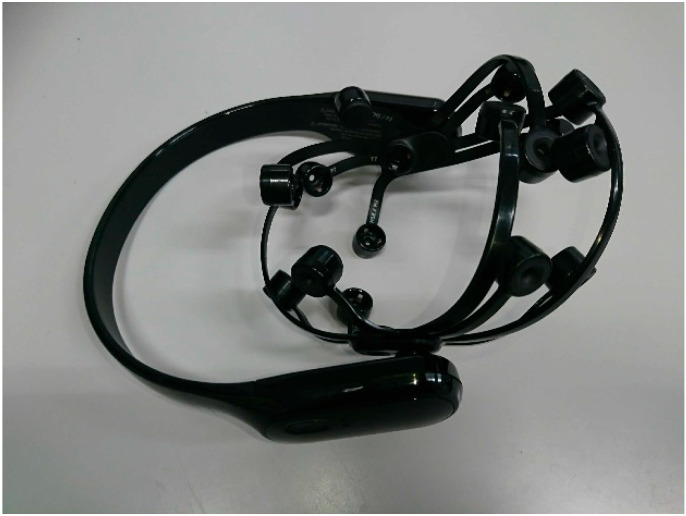
Image of the EEG device used for data collection (EPOC X; Emotiv Inc.).

**Figure 2:**
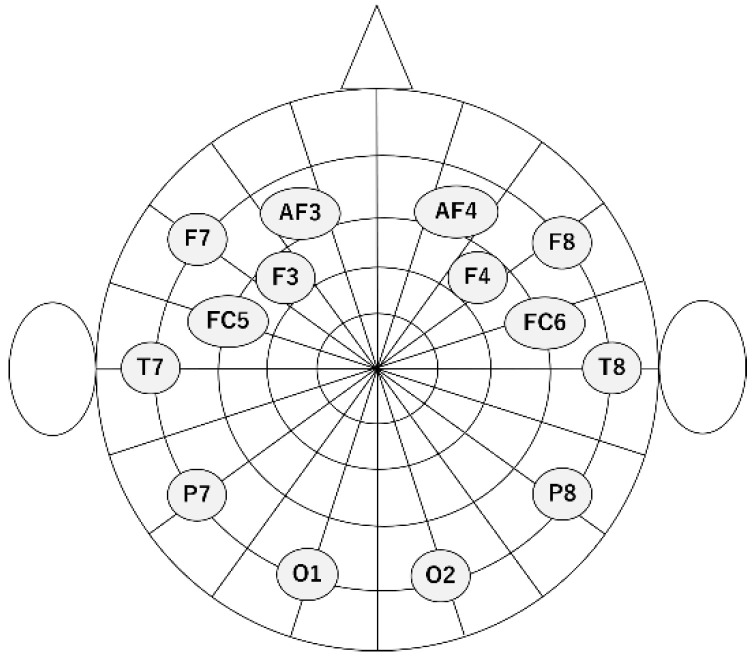
A schematic diagram of the electrode positions.

An EEG device (EPOC X; Emotiv Inc., San Francisco, CA, USA) was used for data collection in our experiment (Figure 1). The sampling rate was set to 256 Hz. The number of electrodes was 14 with the electrode positions at AF3, F7, F3, FC5, T7, P7, O1, O2, P8, T8, FC6, F4, F8, and AF4 according to the international 10–20 system. A schematic diagram of the electrode positions is shown in Figure 2.

Note that the participants were equipped with not only an EEG device but also a photoplethysmography (PPG) device and an electrocardiograph (ECG) device. Both devices are small, lightweight, and can be worn without discomfort, and thus are considered to have little adverse effect on the EEG data. In this paper, we only analyzed the EEG data. The PPG and ECG data will be used for our future research.

### 2.3. Experiment Procedures

**Figure 3:**
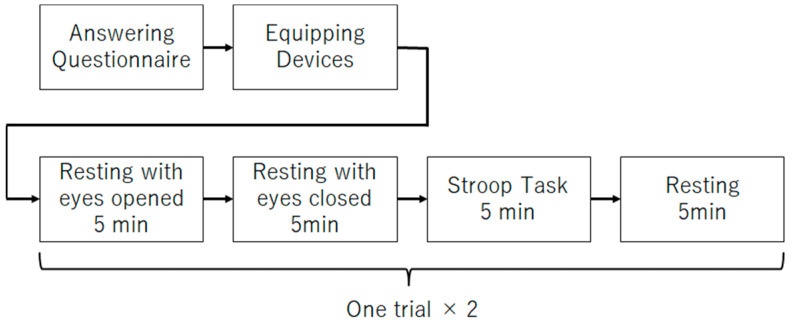
Experiment procedure.

The main experiment procedure (Figure 3) for data collection is described as follows:

(1) Five minutes of rest with eyes open;
(2) Five minutes of rest with eyes closed;
(3) Five minutes of Stroop task;
(4) Five minutes rest;
(5) Repeat (1)–(4) once.

Step (2) of the procedure was performed to obtain the data to be analyzed in this study. In recent years, it has been reported that EEG obtained while the participants are at rest with their eyes closed can be useful in estimating depression. Therefore, this study will focus on the EEG during the rest rather than the EEG during Stroop task. Steps (1) and (3) were performed to obtain data for use in future studies. Step (4) was performed to allow participants to rest. Step (5) was performed to increase the data.

### 2.4. Questionnaire

Participants responded to a questionnaire in which they indicated the name of the disorders they had been diagnosed with and the medications they were taking. If there was no diagnosis of disorders or medication, the participants did not need to fill out any forms and were considered as healthy controls. The names of the mental disorders included in the questionnaire were Anxiety Disorder, Bipolar Disorder, and Depression. The names of developmental disorders were Developmental Disorder, Attention-Deficit/Hyperactivity Disorder (ADHD), Autism Spectrum Disorder (ASD), and Asperger’s Syndrome. Some of the participants described both these mental and developmental disorders. To select depressed patients and healthy controls’ data, the name of disorders filled out in the questionnaire was confirmed.

The question items included in the questionnaire are listed in Table 1. Note that these listed questions are translated from Japanese to English, and are slightly different from the original version due to the translation.

The number of participants divided by genders of the healthy controls and the participants diagnosed with mental disorder are shown in Table 2. Note that several people have been diagnosed with multiple disorders.

### 2.5. Experiment Set-Up and Environment

The experiment was conducted with the participants sitting on a chair facing a desk. The desk was equipped with a PC that guided the participants through the experiment, such as the start and end of the eye-opening rest and the start of responses to the Stroop task, as well as a PC for data collection. During the experiment, participants wore earphones to reduce unintentional auditory stimulation.

**Figure 4:**
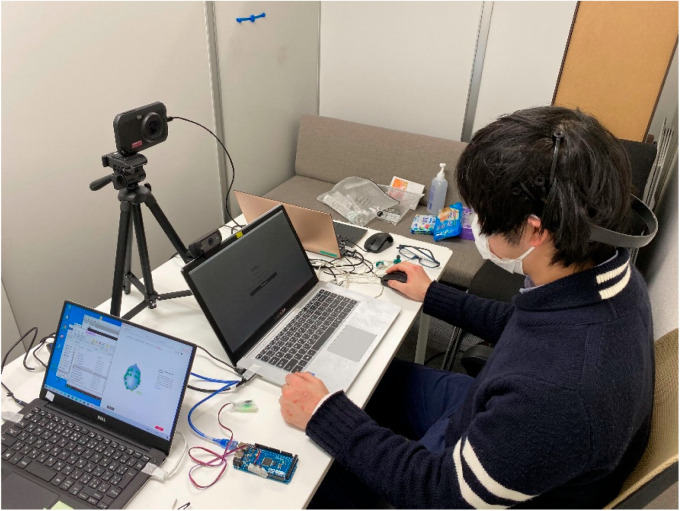
Experimental scene.

The experimental scene is shown in Figure 4. The person sitting on the right is the research participant. The computer in front of the participant displays experiment instructions. Following the instructions, the participant proceeds with the experiment by opening his/her eyes, closing his/her eyes, solving the Stroop task, etc.

## 3. Dataset Construction

**Figure 5:**
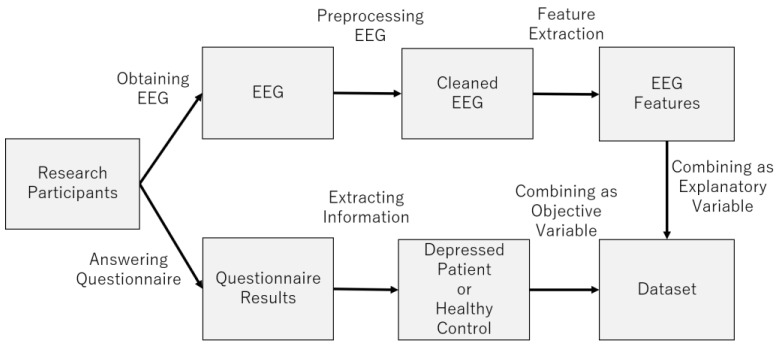
Dataset construction process.

**Figure 6:**
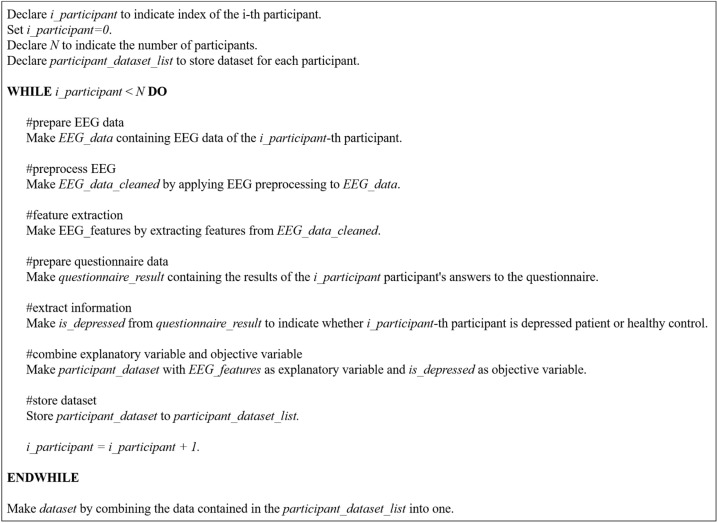
Pseudocode for dataset construction process.

Figure 5 provides an overview of the dataset construction process. The following sections describe the process in more detail (Figure 6).

### 3.1. Preprocessing EEG Data

EEG data usually includes noises due to various factors. In particular, consumer-grade EEG devices are considered to have lower quality and to be more noisy than medical-grade EEG devices. Therefore, noise removal is important to increase the accuracy of depression detection. Thus, we describe the method in detail. To reduce noises, we preprocessed EEG data by EEGLAB [14] and its plug-ins. The data preprocessing steps are as follows: (1) 2 Hz high-pass filter, (2) noise reduction at 50 Hz and 60 Hz, (3) removal of noisy electrode data, (4) Artifact Subspace Reconstruction (ASR), (5) Common Average Reference (CAR), (6) independent component analysis (ICA), (7) ICLabel, (8) removal of noisy independent components, (9) reconstruction of independent components, (9) interpolation of removed electrode data.

In procedure (1), a 2 Hz high-pass filter was used to reduce a noise caused by sweat and low-frequency noise’s negative effect on the independent component analysis (ICA), which is a denoising method to be used later. A *pop_eegfiltnew* function of EEGLAB was used for this process.

In procedure (2), the 50 Hz and 60 Hz frequency bands are affected by humming noise from the AC power supply. To reduce this noise, a *pop_cleanline* function of EEGLAB was used.

In procedure (3), EEG data from electrodes with a lot of noise and from the electrodes that could not obtain EEG disconnected to the scalp are also noises. To reduce these noises as noisy electrodes, the *pop_clean_rawdata* function in the *Clean_rawdata* plugin of EEGLAB was used.

In procedure (4), an ASR algorithm was used to reconstruct the data during the time period when the noise is likely to occur [15]. By reconstructing the noisy data instead of removing it, we prevented the data length from being reduced by the removal. A *pop_clean_rawdata* function in the *Clean_rawdata* plugin of EEGLAB was used for this process.

In procedure (5), EEG data of each research participant was re-referenced by calculating the average value and subtracting it from the EEG data of all electrodes. A *pop_reref* function of EEGLAB was used for this process.

In procedure (6), ICA was used to reduce the noise caused by biological activities such as blinking, eye movement, muscle potential, and heartbeat. By using ICA, EEG data with noise is separated into several independent components.

In procedure (7), the *ICLabel* plug-in [16] of EEGLAB was used to estimate the possibility that an independent component is noisy. The *ICLabel* plug-in removed the components that were not determined to be EEG data with a probability of more than 70%.

In procedure (8), the independent components that were not removed are inversely transformed by ICA. This is expected to extract a clean EEG signal with less noise.

In procedure (9), if some EEG data obtained from noisy electrodes were removed by the process described in above procedures, the removed electrode data were interpolated by spherical splines algorithm [17]. A *pop_interp* function of EEGLAB was used for this process.

### 3.2. Feature Extraction of EEG Signal

EEG indices to be learned by the machine learning model are extracted from the EEG data. The EEG data used in this study were obtained only during the resting state with closed eyes.

Before extracting the EEG indices, the EEG data are divided by sliding a 60 s window every second for data augmentation [18]. This data augmentation reduces the problem of small amount of data. EEG indices are calculated from each of the 60 s of the divided EEG data. The calculated EEG indices are divided into the following four types based on the calculating method: spectral indices, nonlinear indices, left–right asymmetry indices, and functional connectivity indices.

#### 3.2.1. Power Spectrum

The power spectrum is an EEG index used in a wide range of studies as the primary method of EEG analysis [19] and represents the strength of each frequency band. The frequency bands defined in this study are shown in Table 3.

Absolute power was calculated from the EEG data obtained from each electrode for each of the frequency bands listed in Table 3. A total of 224 (14 × 16) features were calculated as absolute power, because the number of electrodes was 14 (Figure 2) and the number of frequency bands was 16 (Table 3). The naming scheme for the features of the absolute power spectrum index was as follows: “<electrode name> absolute <frequency band name>“.

Relative power, which quantifies the relative strength of the 12 frequency bands θ, α, β, γ, Low α, High α, Low β, High β, Low γ, Mid γ, and High γ to the power in the 1–128 Hz band, was also calculated. A total of 168 (14 × 12) features were calculated as relative power, because the number of electrodes was 14 (Figure 2) and the number of frequency bands was 12. The naming scheme for the features of the relative power spectrum index was as follows.”<electrode name> relative <frequency band name>”.

The total number of absolute and relative power features is 392. The MNE-Python library was used to calculate these power spectra [20].

#### 3.2.2. Nonlinear Indices

Nonlinear indices quantify the regularity, predictability, and self-similarity of EEG data [21]. The nonlinear indices used in this study were as follows: Cumulative Residual Entropy (CREn), differential entropy (DiffEn), Shannon Entropy (ShanEn), Spectral Entropy (SpEn), Katz Fractal Dimension (KFD), Petrosian Fractal Dimension (PFD), Sevcik fractal dimension (SFD), Hjorth’s complexity (Hjorth), Hurst exponent (Hurst), Normalized Length Density (nld), Power Spectral Density slope (PFDslope), Relative Roughness (RR), and Standardized Dispersion Analysis (sda). These nonlinear indices were calculated from EEG data passed through bandpass filters for each of the frequency bands shown in Table 3. A total of 2912 (14 × 16 × 13) features were calculated as nonlinear indices, because the number of electrodes, frequency bands, and nonlinear indices are 14 (Figure 2), 16 (Table 3), and 13, respectively. The naming scheme for the features of the nonlinear index is as follows:”<electrode name> <frequency band name> <nonlinear index name”. The Neurokit2 library [22] was used to calculate these nonlinear indices.

#### 3.2.3. Asymmetry Indices

The asymmetry index quantifies the difference between left and right brain activity. There are three asymmetry indices used in this study, which were calculated by calculating the difference (DASM), quotient (RASM), and log-transformed difference (LogDASM) of EEG indices calculated from EEG data acquired from asymmetric electrodes [10,23]. The EEG indices used to calculate asymmetry indices were power spectrum indices and nonlinear indices. A total of 4368 (7 × 16 × 13 × 3) features were calculated as asymmetry indices because the number of symmetrical electrodes, the number of frequency bands, the number of nonlinear indices, and the number of asymmetry indices were 7 (Figure 2), 16 (Table 3), 13, and 3, respectively. The naming scheme for the features of the asymmetry index is as follows: “<asymmetry index name> <Name of the index used to calculate the asymmetry index>”.

#### 3.2.4. Functional Connectivity Indices

Functional connectivity indices quantifies the statistical dependence of time-series data recorded in different brain regions [24]. These indices can analyze the coordination of neuronal activity in the brain [10]. In this study, two electrodes were selected from 14 electrodes and the statistical dependence of the EEG data obtained from these electrodes was quantified. In all cases where two electrodes were selected from 14 electrodes, the functional connectivity indices were calculated, which include coherence (coh), sum of real parts of coherence (cohy), sum of imaginary parts of coherence (imcoh), Phase Locking Value (plv), corrected imaginary plv (ciplv), Pairwise Phase Consistency (ppv), Phase Lag Index (pli), Weighted Phase Lag Index (wpli), debiased estimator of squared WPLI (dwpli), mutual information (mi). A total of 14,560 (C214 × 16 × 10) features were calculated as functional connectivity indices, because the number of combinations to select two electrodes from 14 electrodes (Figure 2), the number of frequency bands, and the number of functional connectivity were C214, 16 (Table 3), and 10, respectively. The MNE-Python library [20] and the Scikit-learn library [25] were used to calculate these functional connectivity indices. The naming scheme for the features of the functional connectivity index was as follows: “<functional connectivity index name> <frequency band name> <first electrode name> <second electrode name>“.

### 3.3. Data Labeling

Label data were generated from the names of disorders obtained from the questionnaire. If the name of the disorder filled in the questionnaire included depression, the label for the data generated from the EEG data of that research participant was set to 1. If the name of the disorder filled in the questionnaire was a blank answer, the label for the data generated from the EEG data of that research participant was set to 0.

### 3.4. Dataset Composition

Each research participant’s EEG data were collected twice during a 5 min period of closed-eye rest. This EEG data were divided by sliding a 60 s window every second. Therefore, about 480 pieces of EEG data are calculated for each research participant. EEG indices used as features were calculated from each of these divided EEG data. Then, the label is set for each of the 480 EEG data. Hence, the amount of data in the dataset is roughly 8160 (480 × 17), which includes data from 17 participants. In addition, each piece of data has a total of 22,232 features, because the number of power spectrum indices, nonlinear indices, asymmetry indices, and functional connectivity indices are 392, 2912, 4368, and 14,560, respectively.

### 3.5. Data-Label Cleaning

To label the data, the results of a questionnaire in which participants describe the name of the disorder diagnosed by the clinician are used. However, incorrect data labeling might occur due to on the subjectivity of patients and clinicians [3,26]. This study addresses this problem by applying Confident Learning (CL) [27,28], a method for cleaning data with incorrect labels.

CL is a method to estimate whether the label data are wrong and to eliminate them [27,28]. To estimate whether the label data are wrong, a model is trained on the training data. This trained model is used to estimate which label the data belongs to. If the estimated probability is greater than or equal to the threshold value$\;t_{j}$, then the true label of the data is assumed to be label $y^{*}$. This threshold value $t_{j}$ is expressed by the following equation.

(1) $$t_{j}=\frac{1}{\left|X_{\tilde{y}=j}\right|}\sum_{X\in X_{\tilde{y}=j}}{\hat{p}}_{j}\left(x\right)$$

where label $\tilde{y}$ is the label attached to the data of the features. x is one piece of data for the features. X is the training data, $\tilde{y}$ is the label, and n is the amount of data. ${\hat{p}}_{j}$ is the probability that one piece of data x belongs to label j, which is estimated by the trained model.

Next, the number of data x in X that are labeled $\tilde{y}$ on the training data and estimated as label $y^{*}$ in the trained model is counted as matrix $C_{\tilde{y},y^{*}}$. The formula for $C_{\tilde{y},y^{*}}\left[i\right][j]$, the i-th row and j-th column of the matrix $C_{\tilde{y},y^{*}}$, is as follows:

(2) $$C_{\tilde{y},y^{*}}\left[i\right]\left[j\right]≔\left|{\hat{X}}_{\tilde{y}=i,y^{*}=j}\right|$$

(3) $${\hat{X}}_{\tilde{y}=i,y^{*}=j}≔\left\{x\in X_{\tilde{y}=i}:{\hat{p}}_{j}\left(x\right)\geq t_{j},j=\arg\begin{array}{c} \min\\ \;k\in M:{\hat{p}}_{k}\left(x\right)\geq t_{k}\; \end{array}{\hat{p}}_{k}\left(x\right)\right\}$$

The matrix $C_{\tilde{y},y^{*}}$ is normalized and the simultaneous probability is calculated as ${\hat{Q}}_{\tilde{y},y^{*}}$. ${\hat{Q}}_{\tilde{y},y^{*}}$ is the following.

(4) $${\hat{Q}}_{\tilde{y},y^{*}}\left[i\right]\left[j\right]=\frac{\left(\frac{C_{\tilde{y},y^{*}}\left[i\right]\left[j\right]}{\sum_{b=1}^{m}C_{\tilde{y},y^{*}}\left[i\right]\left[b\right]}\cdot \left|X_{\tilde{y}=i}\right|\right)}{\sum_{a,b=1,}^{m}\left(\frac{C_{\tilde{y},y^{*}}\left[a\right]\left[b\right]}{\sum_{b=1}^{m}C_{\tilde{y},y^{*}}\left[a\right]\left[b\right]}\cdot \left|X_{\tilde{y}=a}\right|\right)}$$

In the off-diagonal component of this simultaneous probability ${\hat{Q}}_{\tilde{y},y^{*}}$, the class $\tilde{y}$ is searched, whose $\tilde{y}$ is ${\hat{p}}_{x,\tilde{y}=j}-{\hat{p}}_{x,\tilde{y}=i}$ is maximized. The data belonging to the class $\tilde{y}$ are estimated as clean data [27,28]. The CleanLab library [27,29] was used to run this algorithm.

## 4. Model Construction

**Figure 7:**
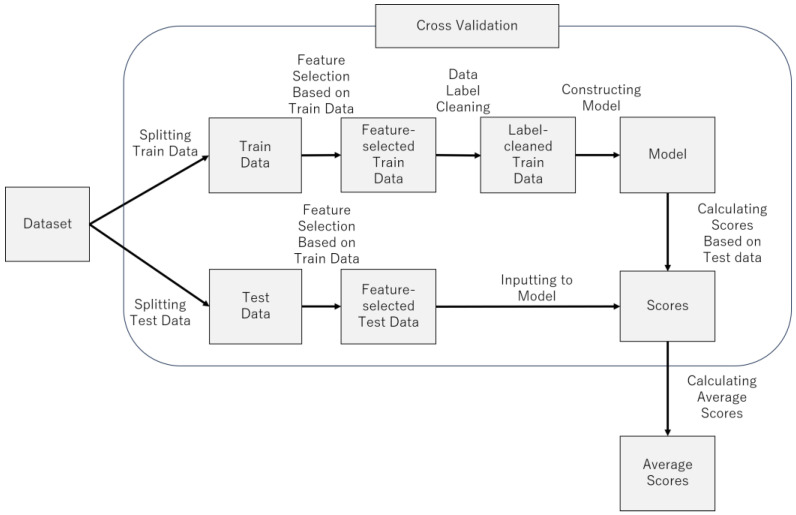
Model construction process.

**Figure 8:**
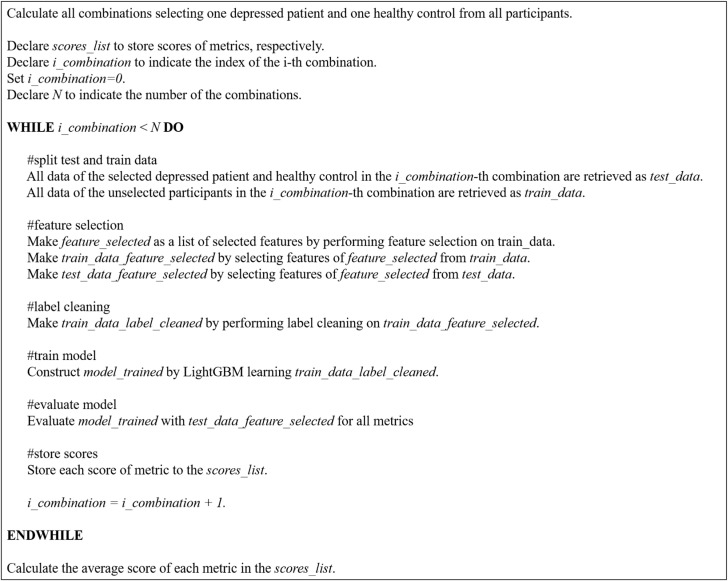
Pseudocode for model construction process.

Figure 7 provides an overview of the model construction process. Figure 8 provides pseudocode for model construction process. The following sections describe the process in more detail.

### 4.1. LightGBM

In this study, we used LightGBM, a gradient learning framework with a decision tree-based learning algorithm, for constructing the model. LightGBM has been improved in terms of memory usage and learning efficiency [30]. It has also been reported that its score is comparable to that of the latest deep learning models on tabular data, such as those constructed in this study [31]. After learning the indices, the model calculates the feature importance. This feature importance is a quantified value of which indices contribute to the estimation. In this study, this feature importance is used for feature selection.

### 4.2. Cross-Validation

Cross-validation was performed to evaluate the generalization performance of the trained machine learning model. To ensure the independence of the training and test data, the training and test data did not include data generated from the same research participant.

Physiological indices such as EEG indices may be similar or dissimilar depending on individual differences. Therefore, if the data of the research participant placed in the training data and the test data are similar, the score is improved, and if not, the score is decreased. Therefore, there will be variations in score depending on the participants placed in the training and test data. These variations in score may lead to higher score by fortunate chance or lower score by unfortunate chance. To prevent such probabilistic problems, all cases of participants assigned to training and test data were considered. Therefore, cross-validation was conducted using the following procedures.

(1) Select one depressed patient.
(2) Select one healthy control.
(3) The EEG data of the participants selected in steps (1) and (2) will be the test data.
(4) The EEG data of the rest of the participants is used as the training data.
(5) The training data are used for training the model, and the test data are used to evaluate the model score.
(6) Steps (1)–(5) are performed for all cases of depressed patients and healthy controls selected in steps (1)–(2).
(7) Calculate the mean of all the accuracies calculated in step (6).

In this study, there were 8 depressed patients and 9 healthy controls, so in step (6), 72 (C19×C18=9×8=72) cycles of model training and score evaluations were performed. In step 7), the average value of these 72 evaluations was calculated.

### 4.3. Evaluation Score

The Macro F1 score was used as the evaluation metric. The Macro F1 score is frequently used in classification and is an evolution of the F1 score used in binary classification [32]. The F1 score is calculated by the following Equations (5)–(7).

(5) $$precision=\frac{TP}{TP+FP}$$

(6) $$recall=\frac{TP}{TP+FN}$$

(7) $$F1-score=\frac{2\times recall\times precision}{recall+precision}$$

In the binary classification of positives and negatives, the variables in Equations (5)–(7) have the following meaning: TP is the amount of data for which the predicted value is positive and the prediction is correct, TN is the amount of data for which the predicted value is negative and the prediction is correct, FP is the amount of data for which the predicted value is positive and the prediction is wrong, and FN is the amount of data for which the predicted value is negative and the prediction is wrong.

The precision is the percentage of data that is truly positive out of the data predicted to be positive. Therefore, this score is emphasized when one wants to reduce the number of missed predictions. The recall is the percentage of data predicted to be positive out of the positive data. Therefore, this score is emphasized when one wants to avoid missing positives. The F1 -score is calculated by the harmonic mean of the score to balance precision and recall. The Macro F1 is the average of the F1 score for each class. The F1 score of each class is calculated with data in one class as positive and data in other classes as negative [32].

In cross-validation, we iteratively construct a model and evaluate these metrics of the model using test data. The test data were all the data of the participants selected in the cross-validation. We finally report the average of the iteratively evaluated scores.

### 4.4. Feature Selection

For training the machine learning model, feature selection is used to improve the score by selecting features that are estimated to be promising for training machine learning model. Four feature selection methods were used in this study: LightGBM feature importance, ReliefF, mutual information, and ElasticNet weight coefficients.

Note that these feature selections were conducted during model construction, which was repeated in cross-validation. In each model construction, the selection was based on the data of all participants assigned to the training data.

#### 4.4.1. LightGBM Feature Importance

The LightGBM feature importance is a quantified value of the degree of contribution in estimating each feature. LightGBM creates multiple decision trees, and the data are classified at the nodes in each decision tree. It is an algorithm that makes a final estimation by voting on the classification results based on those decision trees. The LightGBM feature importance calculation method is similar to Equations (8) and (9).

(8) $$I_{x}=\sum_{n=1}^{N}{\Delta I}_{x,n}$$

(9) $${\Delta I}_{x,n}=G_{Parent}-\frac{m_{Left}}{m}\times G_{Child\;Left}-\frac{m_{Right}}{m}\times G_{Chid\;Right}$$

The definition of Equations (8) and (9) as follows: $I_{x}$ denotes the importance of feature x; N denotes the number of nodes branched by feature x; ${\Delta I}_{x,n}$ denotes the amount of decrease in purity at the nth node branched by feature x; $G_{Parent}$ denotes impurity in the parent node of the nth node; $G_{Child\;Left}$ denotes impurity in the left child node in the nth node; $G_{Chid\;Right}$ denotes impurity in the right child node in the nth node; m denotes the number of data in the nth node; $m_{Left}$ denotes the number of data in the left child node in the nth node; and $m_{Right}$ denotes the number of data of the right child node in the nth node.

At each node in multiple decision trees, the amount of decrease in impurity is calculated by classifying the ground-truth data as in Equation (8). The decrease in the impurity can be interpreted as an increase in the purity, which contributes to the classification and estimation. Therefore, the degree of contribution in estimation is quantified by taking the sum as shown in Equation (9).

This method can be calculated by training with LightGBM. In this study, LightGBM is used as the model; therefore, using LightGBM feature importance as the feature selection method is easy. Thus, this method was used in this study.

#### 4.4.2. ReliefF

ReliefF is an extension of the Relief algorithm [33] developed for feature selection in binary classification [34]. To calculate a score for each feature, Relief rewards the features with close values in neighboring data belonging to the same class and penalizes the features with distant values. Features with large scores are selected as promising for training. ReliefF can be applied to more than two classes, and is known to be useful for noisy data [35]. This feature selection method has been used in previous studies attempting to detect depression by EEG [13,35], and has proven to be highly accurate. Therefore, this method was used in this study. The scikit-rebate library [36] was used to run this algorithm.

#### 4.4.3. Mutual Information

Mutual information is a quantified value of the relationship between two variables. The relationship between the label and the features is quantified, and those with high values are selected as promising features. The Scikit-learn library [25] was used to run this algorithm. The formula for calculating the mutual information content between qualitative and quantitative data is as shown in Equation (10) [37].

(10) $$I\left(X;Y\right)=\sum_{x,y}p\left(x,y\right)\log\left(\frac{p\left(x,y\right)}{p\left(x\right)p\left(y\right)}\right)$$

The definition of Equation (10) is as follows: I(X;Y) denotes the mutual information of X and Y; p(x) denotes the probability of x; p(y) denotes the probability of y. p(x,y) denotes the conditional probability of x and y.

This method is relatively computationally inexpensive and easy to interpret. Therefore, this method was used in this study.

#### 4.4.4. ElasticNet Coefficient

The ElasticNet coefficients are the weights calculated for each feature when training ElasticNet, a linear regression model with a regularization term. When training ElasticNet, the equation to be minimized is as shown in Equation (11) [25].

(11) $$min_{w}\frac{1}{2n}{\left|\left|Xw-y\right|\right|}_{2}^{2}+\alpha \rho {\left|\left|w\right|\right|}_{1}+\frac{\alpha \left(1-\rho \right)}{2}{\left|\left|w\right|\right|}_{2}^{2}$$

The definition of Equation (11) is as follows: X denotes the feature data; y denotes the data of the correct answers; w denotes the weight coefficient of each feature; α denotes a parameter; ρ denotes the regularization term.

By introducing a regularization term, the weight coefficients of features that are not promising for estimation are brought close to zero, and the influence of these features is reduced, thereby improving the model prediction score. Feature selection is performed by removing features whose weight coefficients are close to zero, indicating that they are not promising for the prediction.

There are other methods, such as ridge regression and lasso regression. However, these algorithms have the problem of unstable results when used on data with many features or when there is correlation among features. To reduce this problem, ElasticNet has been improved [38]. This feature selection method has been used in a previous study [39], and its usefulness has been verified. Therefore, this method was used in this study. The Scikit-learn library [25] was used to run this algorithm.

## 5. Results

### 5.1. Questionnaire Results

Some of the questionnaire results used for the analysis are shown in Table 4. Note that the results were translated from Japanese to English, and are slightly different from the original version due to the translation.

### 5.2. Feature Selection Results

We analyzed each of the top five selected features by the feature selection methods. For all feature selection methods, a decrease in score occurred when six features were learned for model training. Therefore, we observed the score of five features selected by each feature selection methods. To analyze these features, the models were trained with each of the indices, and the model accuracies were evaluated with the Macro F1 score. The results are shown in Table 5, Table 6, Table 7 and Table 8.

From all of the Macro F1 scores of all feature selection methods shown in Table 5, Table 6, Table 7 and Table 8, we picked up the top five indices with the highest Macro F1 scorers, consisting of F7 β DiffEn (79.3%), F8 β DiffEn (80.0%), ppc 1–128 Hz F3 AF3 (82.8%), coh 1–128 Hz F3 AF3 (83.01%), and plv 1–128 Hz F3 AF3 (83.7%).

**Figure 9:**
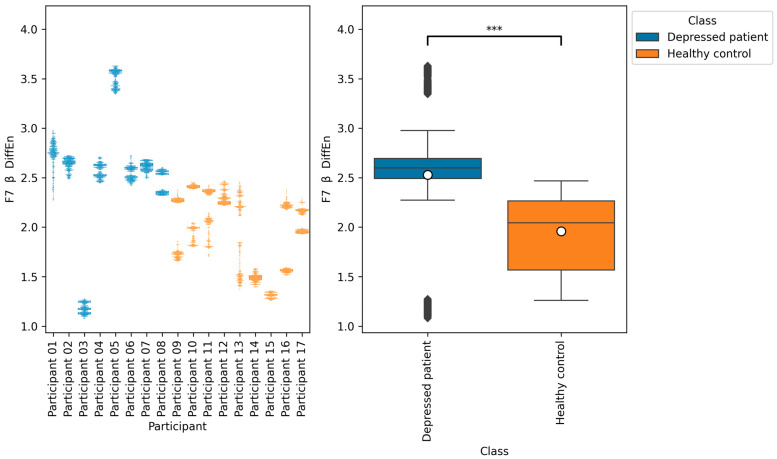
The graph on the left is a bee swarm plot showing the distribution of F7 β DiffEn for each research participant. The graph on the right is a box plot showing the distribution of F7 β DiffEn for groups of depressed patients and healthy controls. The results of the Brunner–Munzel test showed significant differences (*p* < 0.001) in the means of F7 β DiffEn between the depressed patients’ data and healthy controls’ data. In the graph on the left, significant differences were represented as ***. The sample size of the depressed patients’ data was 4097, that of the healthy control’s data was 3856, the significance level was 0.05, and the Cliff’s delta of nonparametric effect size was 0.73.

**Figure 10:**
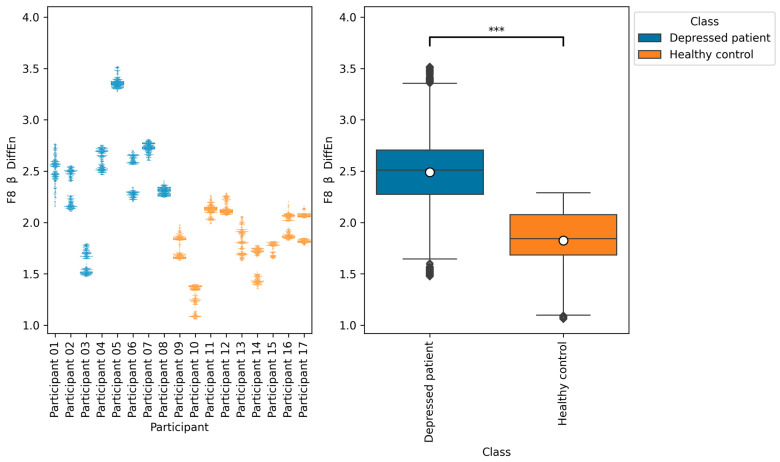
The graph on the left is a bee swarm plot showing the distribution of F8 β DiffEn for each research participant. The left side of this graph shows data for depressed patients and the right side shows data for healthy controls. The graph on the right is a box plot showing the distribution of F8 β DiffEn for group of depressed patients and healthy controls. The results of the Brunner–Munzel test showed significant differences (*p* = 0.00) in F8 β DiffEn between depressed patients’ data and healthy controls’ data. In the graph on the left, significant differences were represented as ***. The sample size of the depressed patients’ data was 4097, the sample size of the group healthy control’s data was 3856, the significance level was 0.05, and the Cliff’s delta of nonparametric effect size was 0.80.

**Figure 11:**
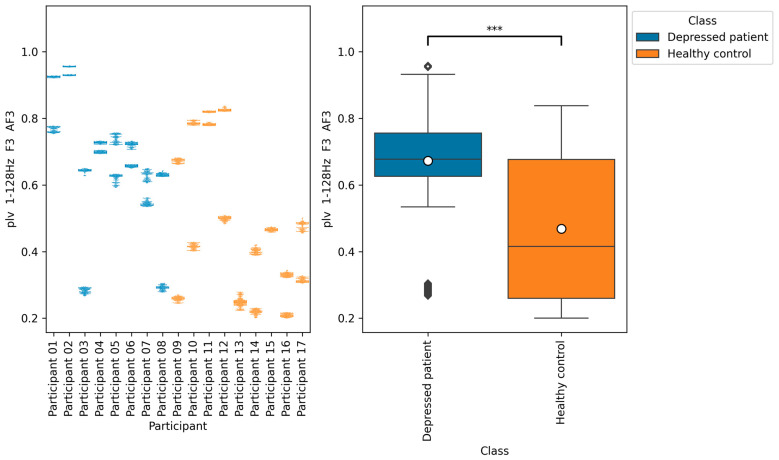
The graph on the left is a bee swarm plot showing the distribution of plv 1–128 Hz F3 AF3 for each research participant. The left side of this graph shows data for depressed patients and the right side shows data for healthy controls. The graph on the right is a box plot showing the distribution of plv 1–128 Hz F3 AF3 for group of depressed patients and healthy controls. The results of the Brunner–Munzel test showed significant differences (*p* = 0.00) in plv 1–128 Hz F3 AF3 between depressed patients’ data and healthy controls data. In the graph on the left, significant differences were represented as ***. The sample size of the depressed patients’ data was 4097, the sample size of the group healthy control’s data was 3856, the significance level was 0.05, and the Cliff’s delta of nonparametric effect size was 0.46.

**Figure 12:**
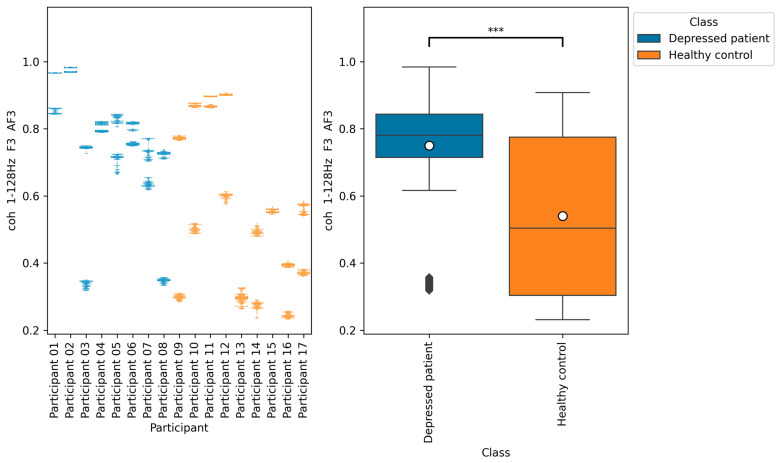
The graph on the left is a bee swarm plot showing the distribution of coh 1–128 Hz F3 AF3 for each research participant. The left side of this graph shows data for depressed patients and the right side shows data for healthy controls. The graph on the right is a box plot showing the distribution of coh 1–128 Hz F3 AF3 for group of depressed patients and healthy controls. The results of the Brunner–Munzel test showed significant differences (*p* = 0.00) in coh 1–128 Hz F3 AF3 between depressed patients’ data and healthy controls data. In the graph on the left, significant differences were represented as ***. The sample size of the depressed patients’ data was 4097, the sample size of the group healthy control’s data was 3856, the significance level was 0.05, and the Cliff’s delta of nonparametric effect size was 0.46.

**Figure 13:**
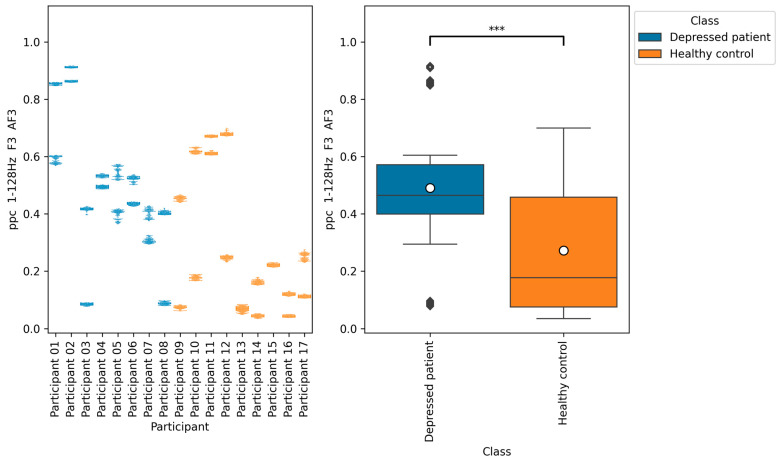
The graph on the left is a bee swarm plot showing the distribution of ppc 1–128 Hz F3 AF3 for each research participant. The left side of this graph shows data for depressed patients and the right side shows data for healthy controls. The graph on the right is a box plot showing the distribution of ppc 1–128 Hz F3 AF3 for group of depressed patients and healthy controls. The results of the Brunner–Munzel test showed significant differences (*p* = 0.00) in ppc 1–128 Hz F3 AF3 between depressed patients’ data and healthy controls data. In the graph on the left, significant differences were represented as ***. The sample size of the depressed patients’ data was 4097, the sample size of the group healthy control’s data was 3856, the significance level was 0.05, and the Cliff’s delta of nonparametric effect size was 0.46.

We further analyzed these indices by plotting a bee swarm and box plot of the data generated for each research participant (Figure 9, Figure 10, Figure 11, Figure 12 and Figure 13) and tested the difference in distribution of each index between the depressed patients and the healthy controls using the Brunner–Munzel test, which does not require normality and homoscedasticity assumptions. Note that some data could have been anomalies, but these data were not removed. This is because anomalies are likely to have been removed in the EEG signal preprocessing procedure. Also, we cannot be sure that they are anomalies.

The results of the Brunner–Munzel tests show significant differences in the means of EEG indices F7 β DiffEn and F8 β DiffEn between depressed patients’ data and healthy controls’ data. In addition, Figure 9 and Figure 10 show that the mean values of F7 β DiffEn and F8 β DiffEn of the depressed patients are higher than those of the healthy controls.

The results of the Brunner–Munzel test showed significant differences in EEG indices plv 1–128 Hz F3 AF3, coh 1–128 Hz F3 AF3, and ppc 1–128 Hz F3 AF3 depressed patients’ data and healthy controls’ data. In addition, the values of these indices tended to be greater in depressed patients than in healthy controls.

**Figure 14:**
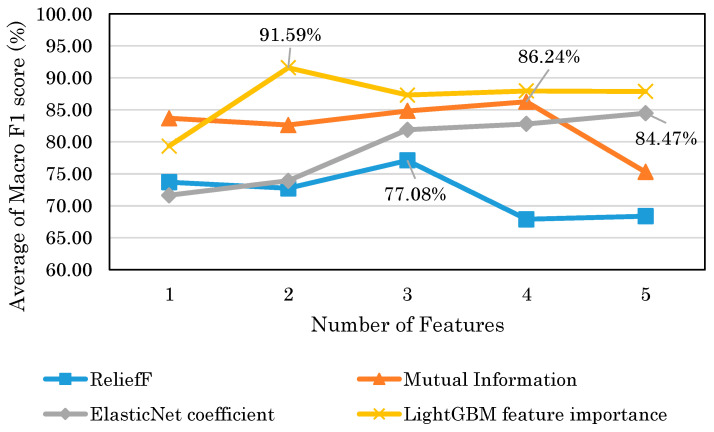
Score displacement of each feature selection method.

Next, we further investigated the score of the models trained with the features selected by the four feature selection methods described in Section 4.4. Figure 14 shows the score displacement indicated by Macro F1 score when the number of features trained into the models is increased according to the descending order of the features estimated to be promising for classification by the feature selection methods. For all feature selection methods, a decrease in score occurs when six features were used for model training. Therefore, we observed the score displacement of the models and plotted up to five features. The numbers in the figure indicate the highest score of each feature selection method. Figure 14 shows that LightGBM has the highest score of 91.59% for feature importance.

## 6. Discussion

In previous studies, models have been constructed to detect depressed patients and healthy controls based on EEG data obtained by medical-grade EEG. On the other hand, in this study, we attempted to construct a model using EEG data obtained by a consumer-grade EEG device. Figure 14 shows that the highest model performance had a Macro F1 score of 91.6%. This result suggests that depression detection is possible with a high score even when EEG is obtained with consumer EEG devices instead of medical EEG devices. The results of this study could facilitate the use of EEG-based depression detection techniques.

According to Table 5, Table 6, Table 7 and Table 8, the results suggest that the selected features by the mutual information tended to be more accurate when trained with only one index. The accuracies of these features range from 76 to 84%, indicating that they were accurate to some extent. However, Figure 14 shows that when multiple features were used, the maximum score of LightGBM was 91.6%, compared to 86.2% for mutual information, indicating that LightGBM feature importance was more accurate. Moreover, in contrast to the mutual information, according to Table 5, the accuracies of each index were low, ranging from 48 to 79%. This may be due to the fact that the variable importance of LightGBM takes into account the interaction between the features, whereas the mutual information measure takes into account the relationship between the features and the target variable. Therefore, the results suggest that the LightGBM feature importance is superior in building models with multiple indices. Mutual information is considered superior for finding stand-alone indices.

According to Table 5, Table 6 and Table 8, the accuracies of F7 β DiffEn and F8 β DiffEn are 79.3% and 80.0%, respectively, which was excellent for the single index only. The results suggest that the indices are promising for classifying depressed patients and healthy controls. However, according to Figure 9 and Figure 10, the data distribution of Participant 03 was different from that of other depressed patients, and the values tended to be low. One of the possible reasons for this is the influence of medication. It has been suggested that a lack of function of the neurotransmitter GABA in brain activity is related to symptoms of depression [40]. According to this participant’s questionnaire result, the participants was taking a GABA-acting medication called Lunesta [41]. Therefore, the effect of this medication on GABA may have caused the data distribution to differ from that of other depressed patients.

According to Figure 11, Figure 12 and Figure 13, the functional connectivity indices calculated from electrodes F3 and AF3 yielded the models with a high score. Therefore, these indices suggest that the indices are promising for classifying depressed patients and healthy controls. These electrodes F3 and AF3 are located in the frontal region, and high functional connectivity in the frontal region of depressed patients has been reported in a previous study [42]. The results of our study were consistent with those findings. However, since some previous studies have reported conflicting results [43,44,45], it is necessary to further study the functional connectivity. Although the above results may be consistent with previous studies, the heterogeneity and differences in study results have been noted [42,43], and we consider that further research is needed.

There are some limitations in this research. The first is the generality of the results. To improve the result of generality and reliability, this study has conducted cross-validation, in which the model is evaluated repeatedly by swapping the training and test data multiple times. However, there exists the possibility of over-fitting only to the data used in this study due to the small number of participants. To resolve this limitation, it is necessary to increase the number of participants and to evaluate the accuracy of the model on multiple datasets. The second is the consideration of the effects of medications, gender, age, and comorbidities. It has been suggested that brain activity can be influenced by medications [13], gender, and age [46]. It has also been suggested that EEG is effective in detecting not only depression, but also developmental disorders [47] and other mental disorders such as bipolar disorder [13]. Therefore, it is possible that the comorbidities of these disorders may reflect something other than depression. For further validation, consideration of these influences would be necessary.

## 7. Conclusions

In previous studies, models have been constructed to detect depressed patients and healthy controls based on EEG data obtained by medical-grade electroencephalographs. However, in this study, we attempted to construct a model using EEG data obtained by a consumer-grade EEG device. EEG indices (power spectrum, complexity, left–right asymmetry, and functional connectivity) quantifying EEG features were calculated from EEG data. For model training, LightGBM, which applies decision trees and gradient boosting, was used. As a selection of promising EEG indices for depression detection, we used the LightGBM feature importance, mutual information, ReliefF, and ElasticNet coefficient. As a result, we proved that the model can be achieved with a Macro F1 score of 91.6% with LightGBM feature importance. In addition, it was indicated that differential entropy in the frequency band beta at electrode F7 and functional connectivity plv, coh, and ppc in the frequency band 1–128 Hz at electrodes F3 and AF3 are promising for the detection of depressed patients and healthy controls.

## 8. Patents

The results of the study reported in this manuscript are patent-pending.

## Figures and Tables

**Table 1:** List of question items in questionnaire.

No.	Question
Q1	What is your gender?
Q2	What is your age?
Q3	What is the name of the disease you have been diagnosed with?
Q4	What medications are you taking?
Q5	What time did you wake up today?
Q6	How have you been feeling in the last month?
Q7	What is your degree of depression, severe fatigue, and irritability? The choices of answer are 4-point ratings: 1 (Almost never), 2 (Sometimes), 3 (Often), 4 (Almost always).

**Table 2:** Number of participants divided by genders in the healthy controls and diagnosed people.

Group	Disorder Name	Number of Male	Number of Female
Participants diagnosed with mental disorder	Anxiety disorder	2	0
	Bipolar disorder	6	2
	Developmental disorder	10	0
	ADHD	10	6
	ASD	17	8
	Depression	7	1
Healthy controls	-	9	0

**Table 3:** Definition of frequency bands.

No.	Frequency Band Name	Symbol	Frequency Bands (Hz)
1	Delta	δ	1–3
2	Theta	θ	4–7
3	Alpha	α	8–12
4	Beta	β	13–30
5	Gamma	γ	30–100
6	Low alpha	Low α	8–9
7	High alpha	High α	10–12
8	Low beta	Low β	13–17
9	High beta	High β	18–30
10	Low gamma	Low γ	31–40
11	Mid gamma	Mid γ	41–50
12	High gamma	High γ	51–100
13	1–30 Hz	1–30 Hz	1–30
14	1–40 Hz	1–40 Hz	1–40
15	1–100 Hz	1–100 Hz	1–100
16	1–128 Hz	1–128 Hz	1–128

**Table 4:** Questionnaire results.

Participant ID	Gender	Age	Diagnosis Name	Medicine
1	Male	26	MDD	Escitalopram Oxalate
2	Male	33	PDD, MDD	N/A
3	Male	29	MDD	Lunesta
4	Male	44	MDD, DD (ADHD, ASD)	Concerta, Tranquilizer, Antidepressants, Muscle Relaxant agent, Stomach medicine
5	Female	27	ADHD, ASD, MDD, OCD	Duloxetine, Bromazepam, Flunitrazepam, Triazolam, Mirtazapine, Lithium carbonate
6	Male	46	MDD, Autism	Tryptanol, Trazodone, Lormetazepam, Zolpidem, Lorazepam, Nitrazepam, Lodopin, Dayvigo
7	Female	46	MDD	Mirtazapine 15 mg, Regtectmg
8	Male	36	MDD, ASD, ADHD	Paxil, Abilify, Intuniv
9	Male	22	N/A	N/A
10	Male	21	N/A	N/A
11	Male	21	N/A	N/A
12	Male	21	N/A	N/A
13	Male	22	N/A	Sinus medicine
14	Male	21	N/A	N/A
15	Male	21	N/A	N/A
16	Male	22	N/A	N/A
17	Male	21	N/A	N/A

**Table 5:** Macro F1 score of each feature selected by LightGBM feature importance.

Rank	Index Name	Macro F1
1	F7 β DiffEn	79.3%
2	F3 θ DiffEn	48.10%
3	P7 θ DiffEn	49.49%
4	coh 1–128 Hz O1 F3	63.56%
5	pli high β O1 F7	70.42%

**Table 6:** Macro F1 score of each feature selected by Mutual Information.

Rank	Index Name	Macro F1
1	plv 1–128 Hz F3 AF3	83.67%
2	coh 1–128 Hz F3 AF3	83.01%
3	ppc 1–128 Hz F3 AF3	82.84%
4	cohy 1–128 Hz F3 AF3	76.86%
5	F7 β DiffEn	79.34%

**Table 7:** Macro F1 score of each feature selected by ReliefF.

Rank	Index Name	Macro F1
1	plv 1–128 Hz F8 P7	73.69%
2	coh 1–128 Hz F8 P7	70.45%
3	cohy 1–128 Hz F8 P7	70.46%
4	ppc high γ F4 F7	61.08%
5	ppc 1–128 Hz F8 P7	72.31%

**Table 8:** Macro F1 score of each feature selected by ElasticNet weight coefficients.

Rank	Index Name	Macro F1
1	F3 γ PFD	71.64%
2	F3 γ nld	74.06%
3	T7 1–100 Hz Hjorth	71.07%
4	cohy mid γ F8 AF3	67.25%
5	F8 β DiffEn	80.01%

## Data Availability

The data used in this study are restricted. This is because we did not obtain consent from the participants to make the data available to the public.
